# Spigelian Hernia: A Clinical Case Report

**DOI:** 10.7759/cureus.46589

**Published:** 2023-10-06

**Authors:** Tran Phung Dung Tien, Nguyen Ngoc Huan, Lam Viet Trung

**Affiliations:** 1 Digestive Surgery, Cho Ray Hospital, Ho Chi Minh City, VNM

**Keywords:** spigelian hernia, open ventral hernia repair, abdominal hernia repair, sublay meshplasty, ventral and incisional hernia

## Abstract

Spigelian hernia, also known as lateral ventral hernia, is a type of hernia arising through the Spigelian aponeurosis. Unlike many other ventral hernias that occur beneath the layer of fat and abdominal skin, Spigelian hernia is situated within the abdominal wall muscles. Spigelian hernia often presents with few symptoms and may exist for a long time without being diagnosed or detected. We report a case of Spigelian hernia causing an intestinal obstruction treated with surgical emergency abdominal wall reconstruction using the sublay technique. Identification and evaluation of cases with the potential for hernia occurrence are crucial for the safety of patients undergoing surgery. Spigelian hernia accounts for 1%-2% of all ventral hernia cases. Currently, there are no reports on Spigelian hernia in Vietnam. However, a few reports on surgical management of Spigelian hernia have been published worldwide, with approaches including laparoscopic and open surgery, and these reports have indicated that abdominal wall reconstruction using the sublay technique is feasible as it is associated with fewer postoperative complications and shorter hospital stays. Here, we describe the case of an 87-year-old woman presenting with swelling and pain in the lower left quadrant of the abdomen. A preoperative diagnosis of Spigelian hernia causing intestinal obstruction was established, and we proceeded with abdominal wall reconstruction using the sublay technique. The patient was discharged three days after surgery without any postoperative complications.

## Introduction

Spigelian hernia was first described by Belgian anatomist Adriaan van den Spiegel in 1645, and, later, Klinkosch reported the first case of Spigelian hernia in 1764 [1]. Spigelian hernia, also known as lateral ventral hernia, is a congenital or acquired defect of the anterior abdominal wall along the semilunar line. It extends through the transversal fascia near the pouch of Douglas. Spigelian hernia is rare, accounting for 1%-2% of all abdominal hernias [2], and there have been no reported cases in Vietnam. Recent reports have described the surgical treatment of Spigelian hernia [3-5], and the open surgical approach for treating this hernia has been accepted worldwide through the guidelines of the European Hernia Society and the International Hernia Society [6,7]. We report a case of Spigelian hernia treated with an open surgical technique using a polypropylene mesh placement and sublay technique.

## Case presentation

**Figure 1:**
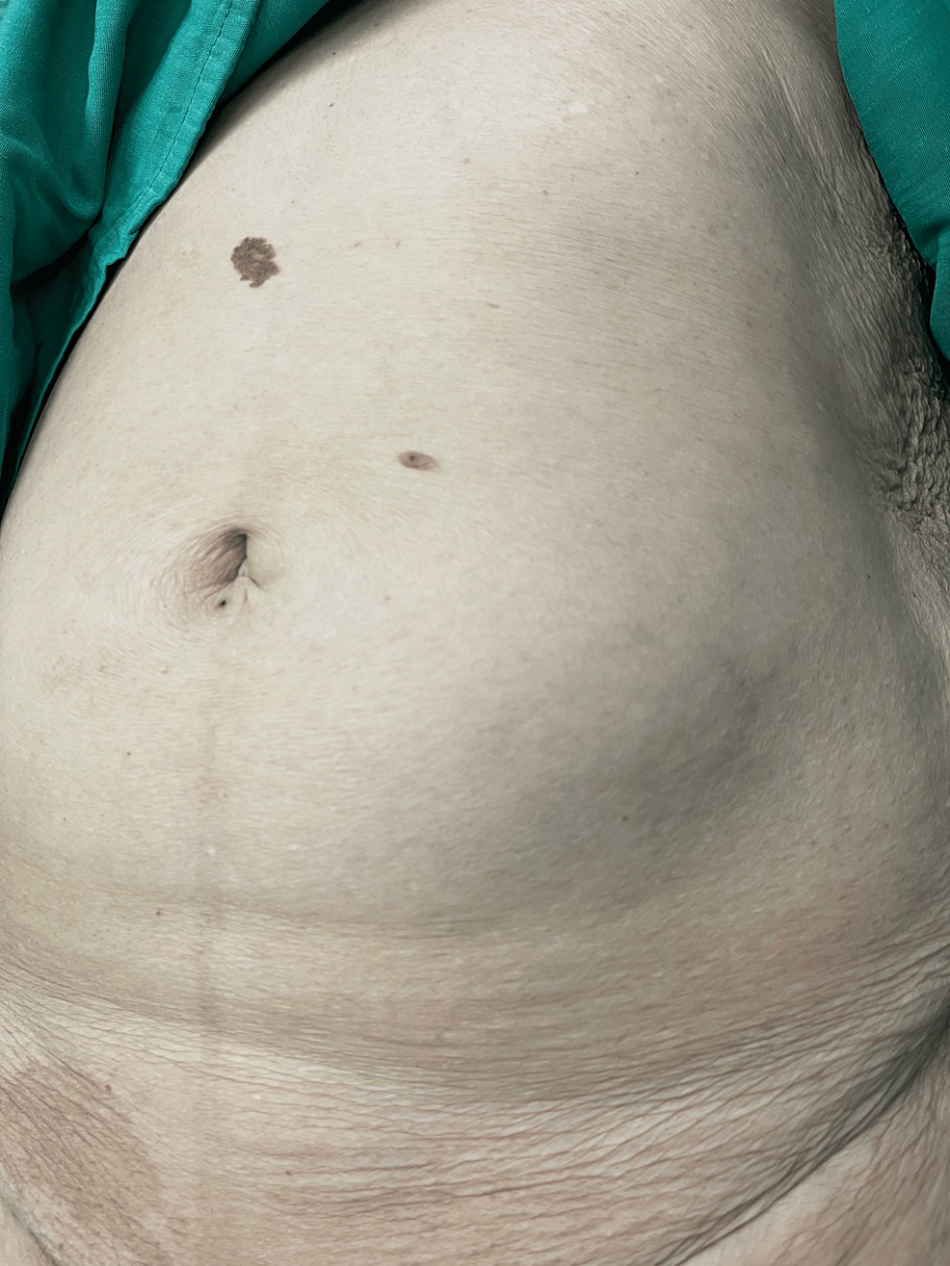
Abdominal wall protrusion in the lower left quadrant.

An 87-year-old woman presented with abdominal pain and a protrusion in the lower left quarter of her abdomen. The protrusion appeared about a year ago, but it had been gradually increasing in size (Figure 1). The patient had abdominal pain for two days, severe pain in the left lower quadrant of the abdomen, intermittent cramping pain, and a gradually distended abdomen.

**Figure 2:**
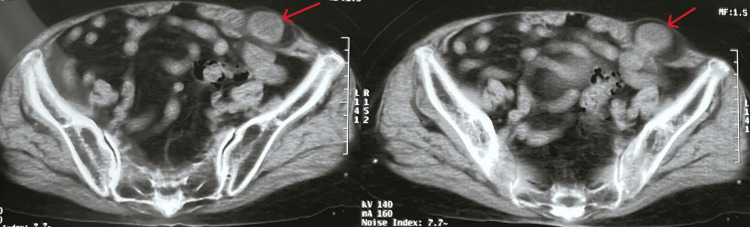
CT scan of the pelvic abdomen showing an image of Spigelian hernia in the lower left abdomen. CT, computed tomography

The patient had no history of abdominal surgery or abdominal trauma. She was slightly overweight with a BMI of 26.5 kg/m^2^. On physical examination, there was a tender protrusion measuring 3 × 4 cm in the lower left quarter of the abdomen. An abdominal CT scan revealed that the small bowel is located anteriorly to the left abdominal wall and extends to the hernia orifice (Figure 2).

The CT scan confirmed the presence of a Spigelian hernia in the lower left quadrant, with the hernia orifice formed through the Spigelian fascia. Prior to surgery, we were able to definitively diagnose a Spigelian hernia causing acute intestinal obstruction. Emergency surgery was performed with the consent of the patient and her family. We used the sublay technique for abdominal wall reconstruction with the placement of a polypropylene mesh. The patient was given general anesthesia, and a 5-cm incision at the hernia site was made.

After dissecting through the subcutaneous layer of the abdomen, we found that the small intestine had herniated through the hernia orifice. We enlarged the hernia orifice and released the small intestine from the hernia. We repositioned the hernia orifice and accurately diagnosed it as an incarcerated Spigelian hernia. We then examined the small intestine and found it to be healthy, with no signs of bowel necrosis. After returning the small intestine to the abdominal cavity, we measured the hernia hole and selected a suitable mesh. The hernia hole was an oval shape, measuring 30 × 40 mm, located on the outer edge of the left rectus muscle. We dissected the posterior sheath of the left rectus muscle and the transversus abdominis muscle on the right side of the hernia hole to create a space. We then sutured the posterior rectus fascia layer and the transversus abdominis muscle with 2.0 absorbable sutures. We used a 6 × 11 cm polypropylene mesh and placed it in the space behind the rectus muscle and the transversus abdominis muscle. We then closed the remaining layers of the abdominal wall with interrupted sutures using 1-0 non-absorbable sutures. The operation time was 100 minutes, with no intraoperative complications. The patient was discharged home three days after surgery, with no postoperative complications.

## Discussion

**Figure 3:**
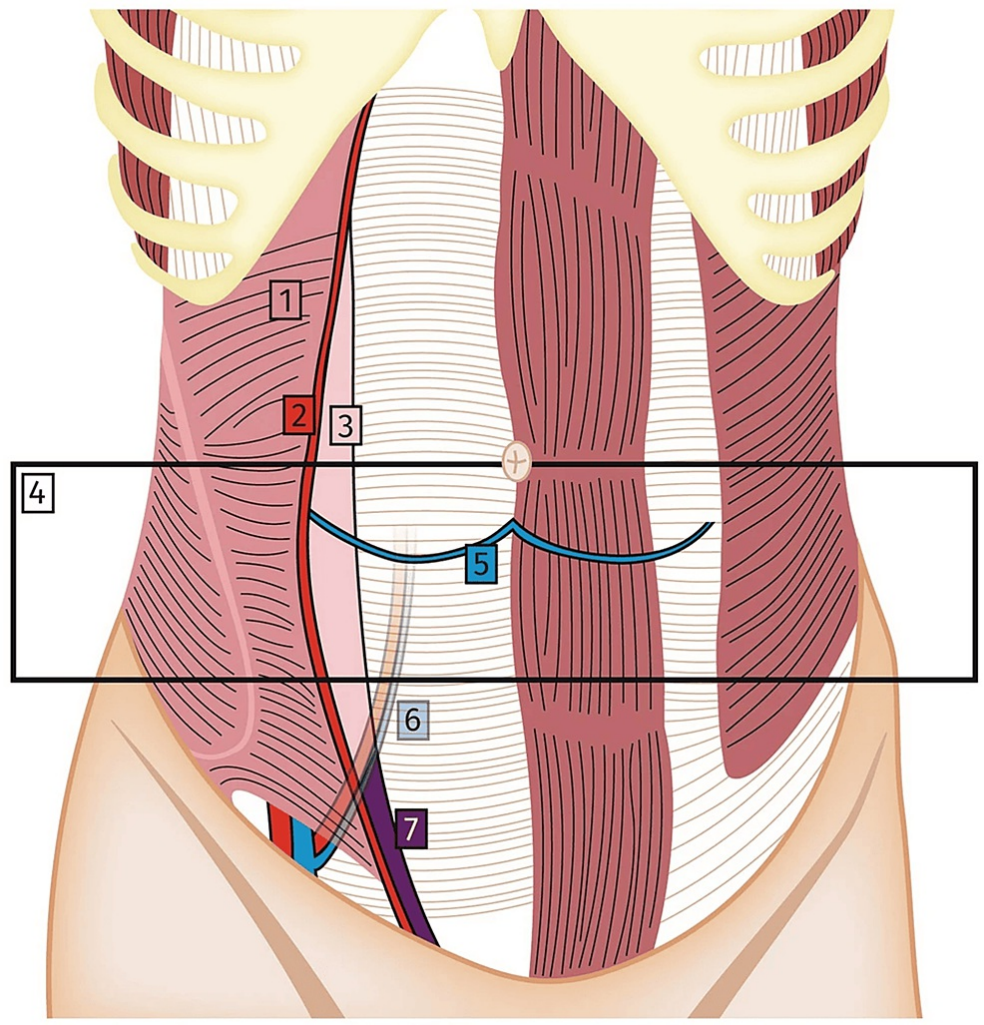
Surgical anatomy of the abdominal wall (coronal view). (1) Transverse abdominal muscle. (2) Semilunar line. (3) Spigelian fascia. (4) Spigelian hernia belt. (5) Arcuate line. (6) Inferior epigastric artery and vein. (7) Low Spigelian hernia area. [2]

Spigelian hernia occurs through the Spigelian aponeurosis, which is the aponeurosis of the transversus abdominis muscle bounded laterally by the semilunar line and medially by the lateral border of rectus abdominis (Figure 3).

This type of hernia usually occurs in people aged 50-60 years, is more common in women than in men, and occurs more frequently on the left side than on the right. Spigelian hernia can occur anywhere along the semilunar line, but most commonly it occurs in the Spigelian hernia belt, a 6-cm wide horizontal area above the interspinous plane, which is the plane joining the anterior superior iliac spines [3]. The exact cause of Spigelian hernia is not known, but its development is related to many factors such as collagen disorders, aging, obesity, rapid weight loss, multiple pregnancies, chronic lung disease, trauma, history of surgery, and reproductive diseases [5]. Most of the described risk factors, including a history of surgery, were not present in our study, although the patient was mildly obese and had four children. The diagnosis of Spigelian hernia based on clinical examination can be difficult. This difficulty may be attributed to its rarity, the absence of classical symptoms, and the lack of clinical experience of the physician. Only around 50% of all Spigelian hernias are diagnosed in the preoperative stage. Most Spigelian hernias have a small defect, and abdominal ultrasound or CT can help establish the diagnosis. In our case description, the effectiveness of an abdominal CT scan after clinical examination allowed for a preoperative diagnosis of Spigelian hernia. We found it necessary to have different differential diagnoses, such as hematoma in the abdomen and abdominal tumor, and to consider performing close clinical diagnostic imaging. Abdominal wall reconstruction surgery using the sublay technique with synthetic mesh placement has been shown to shorten hospital stays and reduce complications and recurrence. This technique has been widely applied in treating Spigelian hernia worldwide [8]. The sublay technique has been around for a long time and is increasingly considered the gold standard for the treatment of abdominal wall hernias [9]. Therefore, we performed open surgery to treat Spigelian hernia using the sublay technique in this case, using a similar procedure and without intraoperative complications.

## Conclusions

In summary, Spigelian hernia is a rare condition with a mass and pain in the lower abdominal wall. Diagnosis and treatment are difficult with beginner surgeons. We have successfully reported the treatment of Spigelian hernia using the sublay mesh and assessed the feasibility of this approach for Spigelian hernia.
